# Supplementary figures and images for: Localized skin inflammation during cutaneous leishmaniasis drives a chronic, systemic IFN-γ signature
Source: PLoS Negl Trop Dis. 2021 Apr 1;15(4):e0009321. doi: 10.1371/journal.pntd.0009321 (PMC8043375; doi:10.1371/journal.pntd.0009321)

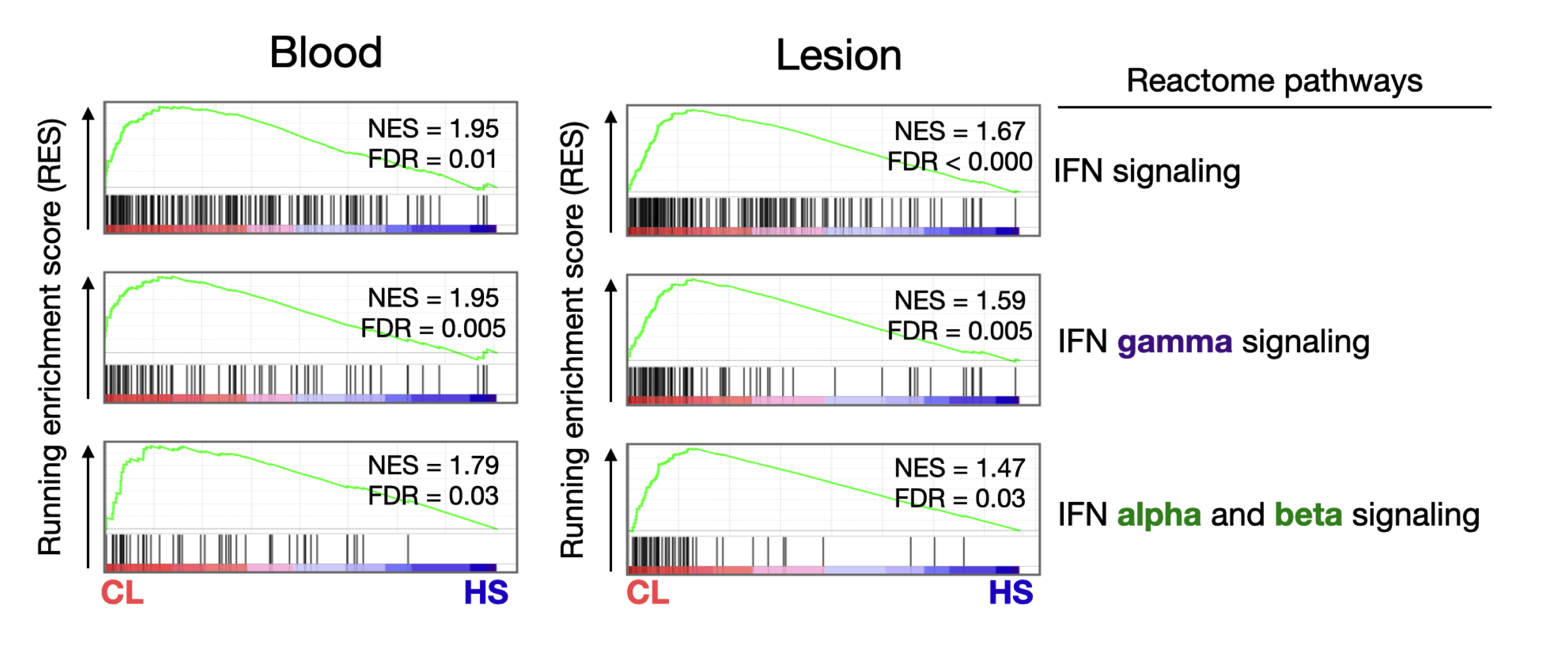

Supplement: S1 Fig — GSEA enrichment plots showing three Interferon-related pathways from the Reactome Pathway Database enriched in the CL peripheral blood (left) and lesion biopsy (right) RNA-seq datasets (IFN signaling, systematic name (sn): M983; IFN gamma signaling, sn: M965; IFN alpha and beta signaling, sn: M973). NES, normalized enrichment score; FDR, false discovery rate. (TIFF) [file pntd.0009321.s001.tiff]

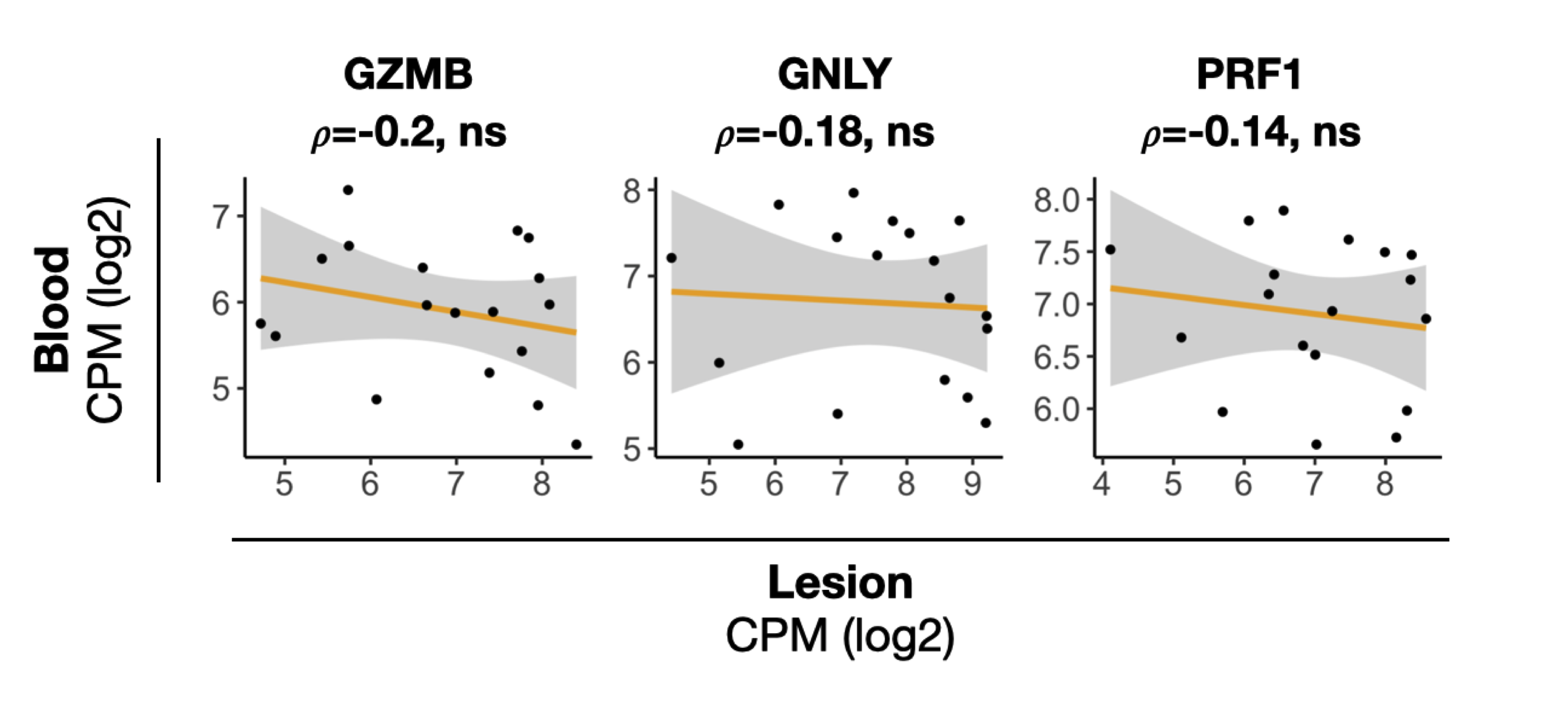

Supplement: S2 Fig — Correlation between the expression of GZMB, GNLY, and PRF1 in peripheral blood and lesions of the same patient. ρ, Spearman’s rho correlation coefficient; ns, non-significant P>.05. CPM, counts per million. (TIFF) [file pntd.0009321.s002.tiff]

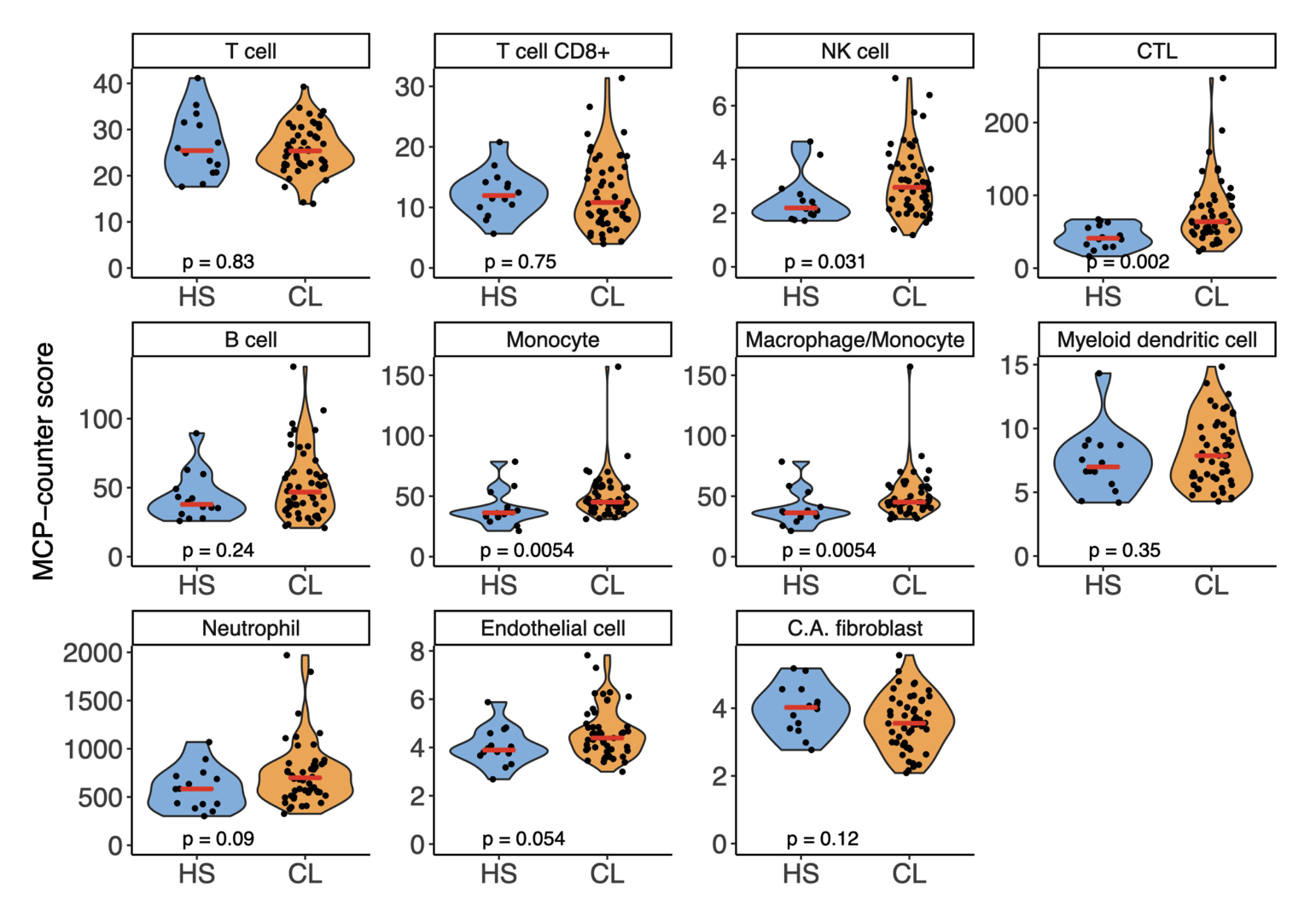

Supplement: S3 Fig — Median of MCP-counter scores for 11 cell types between CL patients (yellow) and HS (blue). Wilcoxon rank-sum test was used for statistical analysis, and P values are represented in the plots. (TIFF) [file pntd.0009321.s003.tiff]

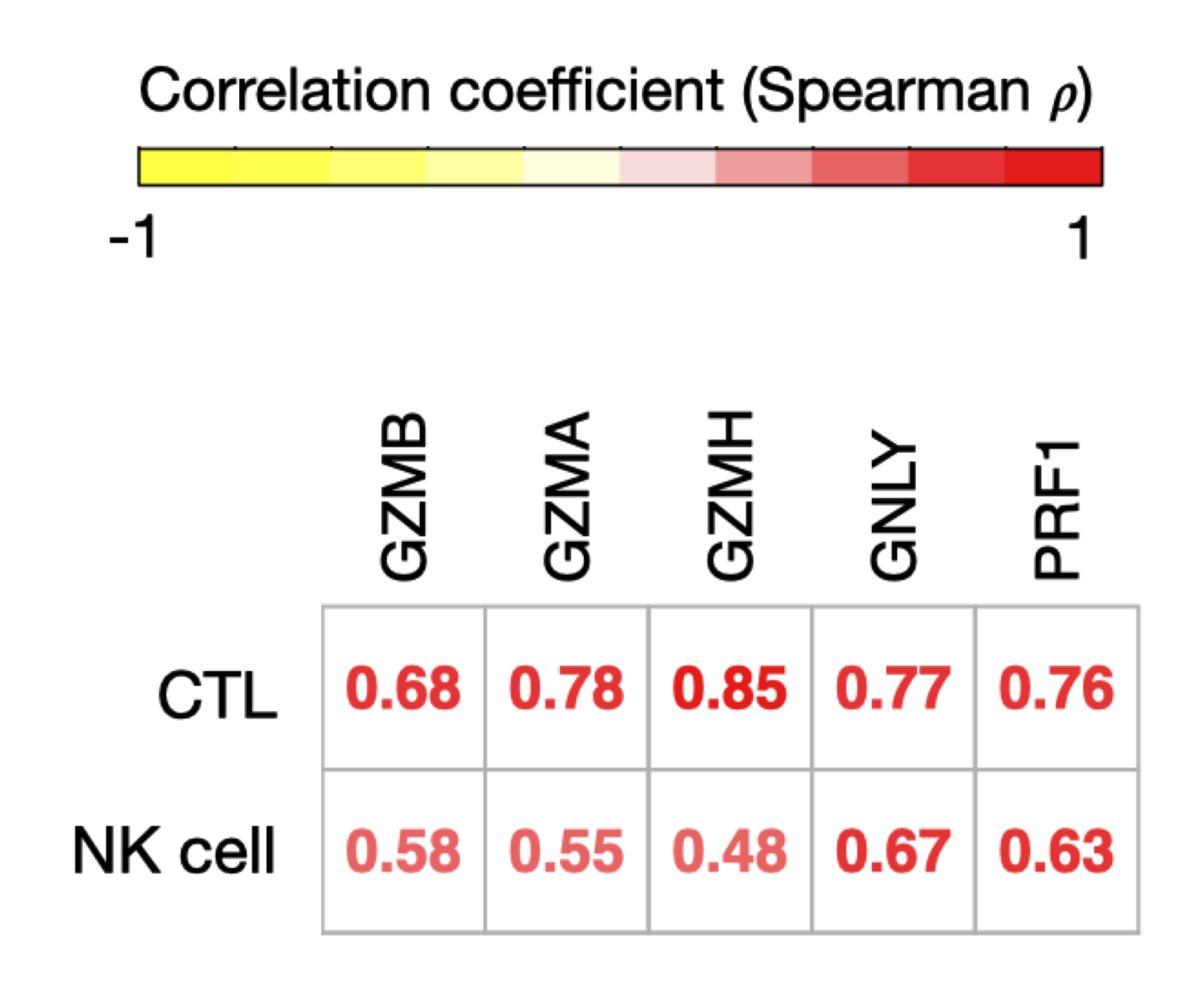

Supplement: S4 Fig — Correlation matrix shows individual correlations between GZMB, GZMA, GZMH, GNLY, and PRF1 genes and the abundance of CTL and NK cells obtained with MCP-counter. The Spearman correlation coefficient ρ is for each correlation is represented in a color scale between -1 and 1 (yellow and red). All correlations included in this matrix were statistically significant, P < .001. (TIFF) [file pntd.0009321.s004.tiff]

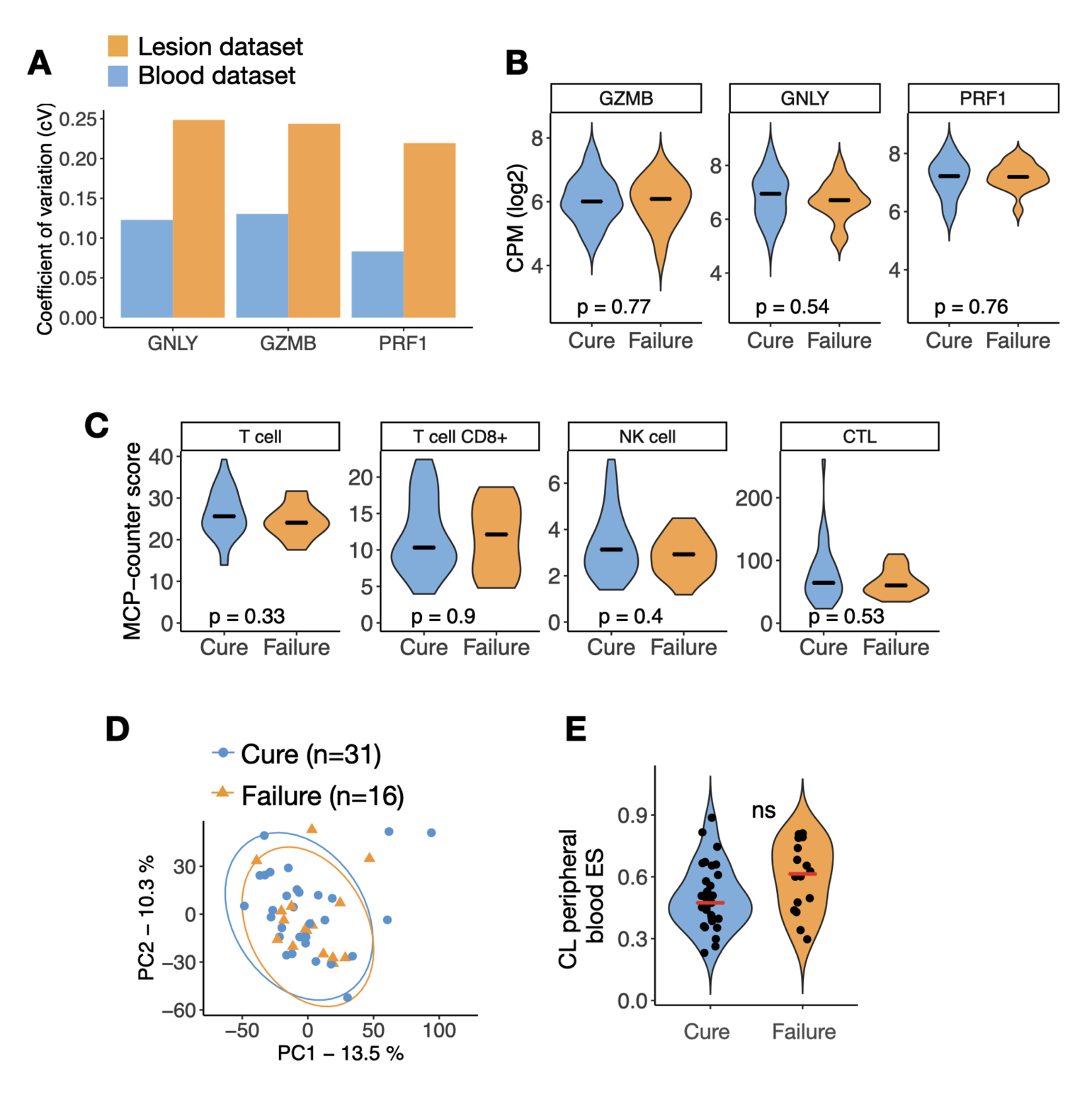

Supplement: S5 Fig — (A) Coefficient of variation (cV) for GZMB, GNLY, and PRF1 expression amongst CL patients in the peripheral blood and lesion biopsy datasets (blue and yellow, respectively). (B) Expression of cytolytic genes (GZMB, GNLY, and PRF1) in the peripheral blood of patients who Cured and Failed the first round of treatment. CPM, counts per million log2 scale. (C) Median of MCP-counter abundance scores of T cells, T CD8+ cells, NK cells, and CTL between CL patients who failed the first round of treatment with pentavalent antimony (n = 16, yellow) and CL patients who cured (n = 31, blue). Wilcoxon rank-sum test was used for statistical analysis, and P values are represented in the plots. (D) Principal component analysis showing principal component 1 (PC1) and PC2 for RNA-seq data from the peripheral blood of CL patients who failed or cured the lesion after the first round of treatment with pentavalent antimony. (B) Enrichment score (ES) of the CL peripheral blood signature (51 genes) by ssGSEA in the blood of CL patient who Failed or Cured. ns, non-significant by Wilcoxon rank-sum test, ns, non-significant, P>.05. (TIFF) [file pntd.0009321.s005.tiff]

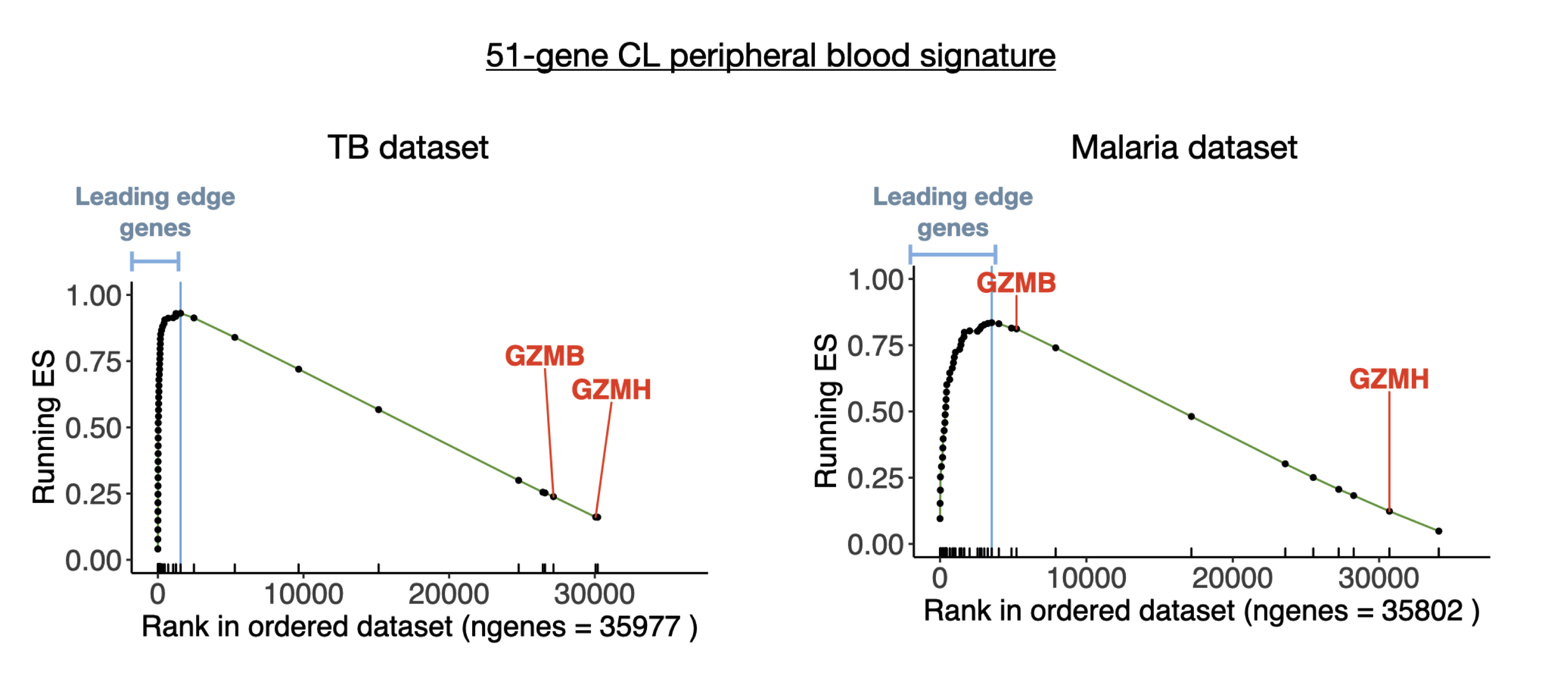

Supplement: S6 Fig — A running enrichment score (ES) plot from a GSEA using the 51 genes in the CL peripheral blood signature as a signature in the TB (left) and uncomplicated malaria (right) datasets. The y-axis shows the running ES from each gene from the peripheral blood leishmanial signature, and the x-axis ranks all the genes in the dataset based on their overrepresentation in the different phenotypes of subjects. The total number of genes in each dataset is indicated in parenthesis. Leading-edge genes included in the left side of the blue vertical line (rank at maximum) are overrepresented in the peripheral blood of patients with active TB and malaria. GZMB and GZMH were highlighted in red the enrichment plots. (TIFF) [file pntd.0009321.s006.tiff]
