# Supplementary material for: Localized skin inflammation during cutaneous leishmaniasis drives a chronic, systemic IFN-γ signature
Source: PLoS Negl Trop Dis. 2021 Apr 1;15(4):e0009321. doi: 10.1371/journal.pntd.0009321 (PMC8043375; doi:10.1371/journal.pntd.0009321)
Supplement: Attachment — Submitted filename: BloodPaperResponsetoReviews.pdf [file pntd.0009321.s008.pdf]

Phillip Scott, PhD
Professor of Immunology
Vice Dean *for* Research & Academic Resources
University of Pennsylvania
School of Veterinary Medicine

February 12, 2021

To the Editors of *PLos Neglected Tropical Diseases,*

We are resubmitting our manuscript entitled "Localized skin inflammation during cutaneous leishmaniasis drives a chronic, systemic IFN-γ signature" with changes suggested by the reviewers. The changes we have made in response to the reviewer's comments are outlined below.

Author's Responses to the Reviewers Questions

Methods

Reviewer #1: The study does not representative a clear advancement in the field, since the major findings were already reported elsewhere for a different outcome of the disease (Visceral Leishmaniasis). However, the objectives are clear and the study is well written, and adds to the literature of Leishmaniasis as a whole.

We agree with the reviewer that our study will add to the leishmaniasis literature. Although a systemic IFN-gamma signature may be expected in VL, where parasitemia is high and systemic, our results show that even highly localized infections with relatively low parasitemia can elicit chronic systemic Th1 inflammation.

Reviewer #2: The manuscript describes a transcriptomic study of whole blood from patients with L. braziliensis CL and healthy controls. A sub study of lesion biopsies was also performed. It is not clear where healthy control biopsies were obtained from in order to define DEGs? Study design is otherwise clear and appropriate to answer the questions posed. Data analysis is thorough and with no obvious errors. No formal sample size calculations were provided but the statistical analysis was appropriate and sufficient for the conclusions drawn.

Thank you for pointing this out. Information on healthy controls has now been added to the Methods Section, "Study Design".

Results

We appreciate the reviewer's comments that the results are clear and the figures of good quality.

----

380 S University Avenue • Room 310 • Philadelphia, PA 19104-4539 • ☎ 215 898-1602• Fax 215 746-2294
http://www.vet.upenn.edu/departments/pathobiology/faculty/ pscott@upenn.edu

Conclusions

Reviewer #1: Yes

Reviewer #2: The manuscript draws the straightforward conclusion that that IFN and cytolytic signatures can be identified int he peripheral blood of CL patients. The IFN signature is perhaps not surprising, given data from other diseases, but it is nice to see. The latter appears more restricted to CL at least in comparison to malaria and TB. Data from a viral infection might have made a nice additional comparator. Public health relevance in terms of identifying biomarkers of disease progression in blood and therapeutic intervention strategies are appropriately discussed.

We appreciate both reviewer's comments. We have included references to viral infections (References 49 and 50)

Editorial and Data Presentation Modifications?

Reviewer #1: (No Response)

Reviewer #2: The authors should address the following minor points:

1. line 96: for completeness the authors should cite PMID: 28959260 where PBMC from healthy and CL patients was compared using microarray.

Thank you for pointing out this reference. We have now added this reference to the Introduction in the suggested position.

2. line 120. The source of healthy control biopsies used to provide data for the lesion DEG analysis (line 234) should be mentioned. In addition, please confirm if parasites from these patients were assessed for LRVs?

The description of healthy control biopsies, which were used in our DEG analysis and other downstream analyses, has now been incorporated into the Methods. The presence of LRVs was not assessed in this study, as we did not obtain isolates from these patients. However, several parasite isolates from Corte de Pedra, Bahia, have previously been tested and they did not contain LRV.

3. line 133: the authors should mention how and why these 17 were selected and whether this was random or associated with disease status.

This information was added to the text. The random selection of peripheral blood from patients and further serum isolation was based on the original sample availability.

4. line 221: Type I and Type II interferons use different receptors -please correct.

This sentence has been corrected, thank you.

5. line 435: blood ISG signatures in blood of VL patients should be cited here (PMID: 31419223)

We added this reference, as well as PMID: 27828962.

380 S University Avenue ♦ Room 310 ♦ Philadelphia, PA 19104-4539 ♦ ☎ 215 898-1602♦ Fax 215 746-2294
http://www.vet.upenn.edu/departments/pathobiology/faculty/ pscott@upenn.edu

6. Discussion: The authors do not mention that Lbb can be found in lymph node aspirates, concurrently with and indeed before lesion development. Hence, it is not only a skin dwelling parasite and LN residence and local immune responses in the LN may contribute to the systemic signature. I think this is worthy of comment and does not detract from the main message here.

We agree and we have added a comment to this effect in the discussion (line 666).

Sincerely,

Phillip Scott, Ph.D.
Professor of Immunology

380 S University Avenue • Room 310 • Philadelphia, PA 19104-4539 • ☎ 215 898-1602• Fax 215 746-2294
http://www.vet.upenn.edu/departments/pathobiology/faculty/ pscott@upenn.edu
