# Supplementary material for: Localized skin inflammation during cutaneous leishmaniasis drives a chronic, systemic IFN-γ signature
Source: PLoS Negl Trop Dis. 2021 Apr 1;15(4):e0009321. doi: 10.1371/journal.pntd.0009321 (PMC8043375; doi:10.1371/journal.pntd.0009321)
Supplement: Attachment — Submitted filename: Review.docx [file pntd.0009321.s007.docx]

In the present study, Amorim et al. expand their work on immune responses to Leishmania parasites, identifying that the ISG and cytolytic transcriptional signature are found not only in the patient’s lesion sites (as previously described), but also in their blood. Although this study does not represent a substantial advancement in the field (the main set of data has been shown in other Leishmaniasis outcomes), these results add to the literature of immune regulation upon Leishmania infection, shedding light into important immunological and pathological aspects related to the disease. The study is well performed and the manuscript is well written. However, I do have some suggestions to improve the message of the paper:

**Major issues:**

The authors should be more careful when stating that type II IFN are more important than type I for their observed transcriptional profiles. The second is induced more acutely than the first, and their different timing/concentrations in the skin could differentially affect inflammation both in the lesion and systemically. Therefore, with no further sets of data, rather than only RNA-seq from patients, it is difficult to conclude that type I IFN are not as important as type II – I suggest reframing of this statement (also the title of the paper), always reinforcing that both could play an important role in the induction of the ISG transcriptional profile.

The authors claim, both at the introduction and discussion, that ISG and cytolytic transcriptional programs are associated with increased inflammasome activation, which might play a deleterious role to the host in the context of cutaneous Leishmaniasis. It would be nice to show if inflammasome-associated molecules, such as Il1b, Il18, NLRP3, Casp1, Casp11, etc, are differentially modulated in the blood of patients compared to healthy volunteers, and also making this comparison between infected patient’s blood and lesion. Moreover, the authors should be more careful on their statements about the role of the inflammasome in cutaneous Leishmaniasis, since several studies have demonstrated a protective role under some conditions – which should not be neglected just because it possibly does not cope well with the authors current hypotheses.

**Minor issues:**

-Introduction, line 75: add a comma (,) after “infection”.

-Introduction, line 78: “memory T cells and contribute to protection [9,10].”. I suggest: “**memory T cells, contributing to protection against the parasite** [9,10].”

-Introduction, line 80: Do the authors mean “unbiased”, instead of “untargeted”?

-Introduction, line 85: “a blood 4-gene signature”. What does this “4” mean?

-Results, line 207: “**Fig 1. Transcriptional profiling identifies an Interferon-stimulated gene (ISG) signature**..”. Please include “type-II” before “Interferon”. It is important to keep it clear that the authors claim that IFN-g, rather than alfa/beta, are involved in this transcriptional profile. Please correct it all along the text (if it ever happens again).

-Results, line 228-229: “**These data suggest that the ISG signature in the blood of CL patients may be driven by IFN-γ.**”. If the vast majority of genes are induced by both type I and II IFNs, and given the important role of both during infections, I don’t believe the authors should make this statement if they do not have any further evidence for a major role of gamma, rather than type I-IFN, in this context.

-What the authors call “yellow” in some legends/figures, I see as orange.

-Discussion, line 459: “**On the other hand, the presence of low levels of IFN-γ leading..**.”. I guess the authors wanted to say “high” instead of “low”?
